# Cases

**Published:** 1891-12

**Authors:** J. F. Winchester

**Affiliations:** Laurence, Mass.


### CASES.

---

### RABIES.

By J. F. Winchester, D. V. S.

Laurence, Mass.

During the week of July, 1890, a bull terrier dog, owned here in town, was bitten by a dog that was running amuck through the highway. The bite was not a severe one, and no special notice was taken of it, but the owner asked me within a few days what was the chance of his dog becoming rabid.

The result of the opinion I expressed was that the animal was under close confinement for a period of about three months, and as he remained in an apparently normal condition, the restriction was removed.

Nothing was observed abnormal until the middle of May, 1891, and on the 17th he was excitable and irritable, and bit a dog that had been his companion for some time. The wound was made over the eye, and had the appearance of being made with one tooth. As a result of that he was again chained in the hay loft, and then began a series of symptoms that would cease only from exhaustion, or the presence of the groom. Barking, tearing anything within reach, breaking windows and sash, and at one time broke his chain. When approached by the groom he would stop and crouch with only quick breathing to be noticed. This condition lasted three days, and he was found dead one morning.

Post mortem : Rigor mortis well marked. The abdominal viscera normal with the exception of the contents of the stomach, which consisted of foreign matter.

The organs in thoracic cavity normal. The blood vessels under the pia mater in the convolutions of the brain were congested, and also the plexi in the three ventricles.

The dog that was bitten May 17 died June 12, having been sick a few days, and the owner told me he had the same actions as the dog that bit him. Prescribed during his sickness.

### Peritonitis and Abnormal Spleen.

Oct. 1. Castrated chestnut colt 17 months old. The manipulation easy and soon over. Colt got up all right, no bleeding, put in box stall, tied up, given hay to eat.

Oct. 3. Visited the colt. He was bright, active, good appetite, full of life, and it required some tact to open the scrotum, which was done, and the usual amount of serum was found.

Oct. 12. Was called and found colt dead at noon.

History : Saturday, the 10th, he was apparently all right, eating well and good spirits, not any unusual degree of swelling of scrotum, the openings discharging well. Sunday the 11th, did not feel well, but ate until evening. No marked pain, but his head was down and dull. Monday, the 12th, up and down during the morning, but no pawing or acute pain, and at noon he died without a struggle.

Post Mortem : Chestnut gelding 17 months old. Not bloated, body warm, well marked rigor mortis, scrotum somewhat swollen, holes open and discharging apparently normal pus. Removing abdominal muscles found the peritoneum inflamed, and opening the same, serum poured out copiously. The small colon was blushed light red in color, the same with the large colon. The small intestines were very red, of deep carmine color, and peritoneum dark red, and the blood vessels in the peritoneum were filled and well defined.

On the right side and under the stomach found an organ, shaped like the spleen, weighing about three pounds, dark blue in color, spongy texture; the capsule on side next stomach ruptured and covered with coagulated fibrin.

On left side and underneath (on top, as animal was on its back) found a spleen, weighing about two pounds, normal in shape, color and texture.

The rest of viscera apparently normal.

### Peritonitis and Death as a Result of Rupture of the External Coat of the Stomach.

History : Animal sick all night with colic. Called to see the horse in morning, found animal down, pulseless, cold sweat, and diagnosed peritonitis with the prognosis of death within a short time.

Post mortem twenty-four hours after death. Black roan gelding. Tympanitis well marked. Removed the abdominal muscles, found peritoneum dark in color, blood vessels well dilated, and a large amount of straw-colored serum in abdominal cavity. The large and small colon were normal in color with the exception of a slight light red blush, which was also found on the small intestines.

The stomach was distended and a rupture of the external coat along the large curvature for nearly two feet. The muscular fibers of the middle coat were separated and well defined. The rest of viscera normal.

### Fistula of Steno's Duct.

Last spring the owner of the horse in question noticed that the animal did not take the bit as kindly as had been his habit, and acted as if his mouth was sore. Within a short time the horse was brought to me, and an examination of the buccal cavity disclosed that the third upper molar was diseased and the outside edges were very pointed ; this condition was remedied, and the animal returned to work with apparent relief.

Two or three weeks after this the owner noticed an enlargement on the left side under the jaw bone, which gradually increased in size until it broke, and a continual stream of clear, thin fluid escaped. This condition the owner tried in vain to control for about three weeks, and then the animal was again brought to my notice.

At once recognizing the trouble, I began to look for the cause, and examining the mouth found that the mucus membrane on the left side of the mouth opposite the third upper molar was corrugated and inflamed. Thinking that the cause of the trouble might originate there I tried to pass a catheter while the animal was standing, but failed to accomplish the feat. Sent the horse home, and had him prepared for casting, and the catheter was passed into the duct down as far as where it runs under the bone and then the animal was allowed to get up.

The opening under the jaw was kept clean, and a strong solution of carbolic acid applied to the part several times a day.

The result of this was that the discharge stopped after a short time, and at time of writing the animal is apparently all right, with the exception that the course of the duct is well defined back to the gland.

